# Prevalence and Predictors of Mortality for Older Adults Referred to a Hospital Avoidance Program

**DOI:** 10.1093/geroni/igaa057.456

**Published:** 2020-12-16

**Authors:** Shahzad Gul, Megan Freund, Robert Sanson-Fisher, Matthew Clapham, Penelope Webster

**Affiliations:** 1 John Hunter Hospital, Hunter New England Area Health Services, Cameron Park , New South Wales, Australia; 2 Health Behaviour Research Collaborative, School of Medicine and Public Health, Faculty of Health and Medicine, University of Newcastle, Callaghan, New South Wales (NSW), Australia, New Lambton Heights, New South Wales, Australia; 3 Health Behaviour Research Collaborative, School of Medicine and Public Health, Faculty of Health and Medicine, University of Newcastle, Newcastle, New South Wales, Australia; 4 Hunter Medical Research Institure, Hunter Medical Research Institure, New South Wales, Australia; 5 Community Acute Post-Acute Care, Hunter New England Local Health District, NSW, Australia, New South Wales, Australia

## Abstract

A cross sectional retrospective data linkage study of older adults discharged from local hospital avoidance program between January 2017 and January 2018 was undertaken (N=286; mean age 80.5 years). The prevalence of death at 3 months, 6 months, 12 months, 18 months and 33 months was calculated. Patient demographic characteristics associated with participant’s risk of mortality at 33 months after discharge was examined using Cox multivariable regression. Patient demographic and health characteristics associated with participant mortality within 12 months of discharge was examined using multivariable logistic regression for patients with complete health characteristic data (n=195). The mortality prevalence was 17% at six months and the cumulative prevalence at one year, 18 months and 33 months post discharge were 24%, 29% and 36% respectively. Patient demographic characteristics associated with participants’ risk of mortality at 33 months after discharge were gender, age and household arrangements. Health and demographic characteristics associated with mortality within 12 months of discharge were lower cognition, increased burden of comorbidity, decreased physical function, a weight less than 55 kilograms, older age and male gender. These results indicate that a significant proportion of people attending a hospital avoidance program are likely to be entering into the final year of their life. This suggests that hospital avoidance programs should routinely identify patients who are likely nearing end of life, and support advance care planning for this patient group.

